# Chitosan-Based Nanoparticles for Nose-to-Brain Drug Delivery: A Real Path toward Effective CNS Therapy?

**DOI:** 10.1021/acsbiomaterials.6c00137

**Published:** 2026-04-23

**Authors:** Lorena R. Riani, Gustavo F. B. Seno, Dominique M. Silva, Cibele R. Toledo, Mayara R. B. Paiva, Júlia S. Santos, Rodrigo L. Fabri, Frederico Pittella, Guilherme D. Tavares

**Affiliations:** † Postgraduate Program in Pharmaceutical Science, 28113 Federal University of Juiz de Fora, Juiz de Fora, Minas Gerais 36036-900, Brazil; ‡ Faculty of Pharmacy, Federal University of Juiz de Fora, Juiz de Fora, Minas Gerais 36036-900, Brazil; § Department of Pharmacology, Institute of Biological Sciences, Federal University of Juiz de Fora, Juiz de Fora, Minas Gerais 36036-900, Brazil; ∥ Department of Biochemistry, Institute of Biological Sciences, Federal University of Juiz de Fora, Juiz de Fora, Minas Gerais 36036-900, Brazil

**Keywords:** CNS disorders, intranasal delivery, nose-to-brain, nanoparticles, chitosan

## Abstract

Treating central nervous system (CNS) disorders remains a major clinical challenge. The blood–brain barrier (BBB), systemic toxicity, and first-pass metabolism are key obstacles. These factors limit the effective drug delivery to the brain. Intranasal administration has emerged as a noninvasive strategy to bypass the BBB. This approach enables direct drug delivery to the brain through the olfactory and trigeminal nerve pathways, commonly referred to as nose-to-brain (N2B) delivery. In this context, chitosan (CS), a biocompatible and mucoadhesive polysaccharide with permeation-enhancing properties, has gained significant interest as a functional material for nanoparticle (NP) engineering. CS-based or CS-coated NP can prolong the residence time on the nasal mucosa and facilitate drug transport to the CNS. This review provides a comprehensive overview of recent advances in CS-based NP for N2B drug delivery across a range of CNS disorders, including neurodegenerative, neuropsychiatric, neoplastic, and infectious conditions. Particular attention is given to formulation strategies, mechanistic insights, and preclinical outcomes. Recent patent applications are surveyed to underscore the translational potential and commercial interest in this technology. Collectively, CS-based NPs effectively address major therapeutic barriers, establishing a transformative and innovative platform in CNS drug delivery.

## Graphical abstract

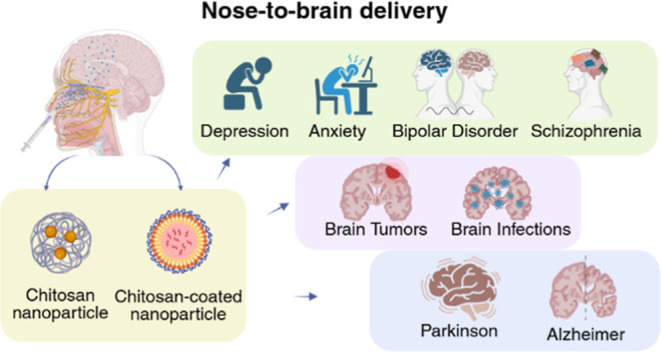

## 1 Introduction

Central nervous system (CNS) disorders constitute one of the leading causes of disability and mortality worldwide. Neurological conditions currently affect an estimated 3.40 billion people, representing 43.1% of the global population. Over the past three decades, both mortality and overall health burden have increased by 39% with this burden projected to increase significantly in the coming decades.^1,2^

This trend is intimately linked to the progressive aging of the global population, which is accompanied by a marked increase in the incidence of neurodegenerative diseases such as Alzheimer’s and Parkinson’s,^3^ as well as neuropsychiatric disorders including depression, anxiety, schizophrenia, and bipolar disorder (BD).^4^ In parallel, the management of CNS tumors and infections continues to pose substantial clinical challenges.^5,6^ Collectively, these conditions underscore the need for effective therapeutic strategies targeting the CNS. Despite extensive investment in drug development, most therapeutic agents exhibit limited clinical efficacy due to their inability to adequately cross the blood–brain barrier (BBB), an evolutionarily conserved structure that restricts the passage of xenobiotics into the brain parenchyma.^7^ As a result, many CNS drugs suffer from poor bioavailability, high systemic toxicity, short half-life, and frequent off-target effects, necessitating high doses and frequent administration schedules.^8,9^

Alternative delivery approaches that can bypass or overcome the BBB have become a focus of scientific research, such as noninvasive delivery strategies.^10^ Among them, the intranasal route has emerged as a particularly promising approach for direct drug delivery to the brain, leveraging the anatomical and physiological connectivity of the nasal cavity with the CNS via the olfactory and trigeminal pathways.^11^ This approach, often referred to as nose-to-brain (N2B) delivery, enables rapid onset of action, reduces systemic exposure, and enhances therapeutic targeting.^12^ Notably, the clinical feasibility of this route is supported by the approval of several intranasal formulations currently on the market for migraine (e.g., zolmitriptan, Sumatriptan), opioid overdose (e.g., naloxone), epilepsy (e.g., diazepam, midazolam), and depression (e.g., esketamine).^13^

Importantly, the intranasal route still presents several challenges that may limit its efficiency. Rapid mucociliary clearance in the nasal cavity, combined with enzymatic degradation within the nasal mucosa, significantly reduces the drug residence time and hinders absorption. Importantly, although the intranasal route offers a promising noninvasive pathway for N2B delivery, it still presents several physiological and formulation-related challenges that may limit its efficiency. Rapid mucociliary clearance in the nasal cavity, together with enzymatic degradation within the nasal mucosa, can significantly reduce drug residence time and hinder absorption.^14^ In addition, particle size plays a critical role in determining nasal deposition, mucosal penetration, and subsequent transport to the brain, with optimal sizes typically in the 100–200 nm range, whereas suboptimal sizes may lead to rapid clearance or limited permeation.

Furthermore, the physicochemical stability of formulations, including resistance to aggregation, degradation, and premature drug release, is essential to ensure consistent performance and therapeutic efficacy.^16^ Therefore, the rational design of N2B delivery systems must carefully consider these factors to optimize drug transport across the nasal mucosa and improve brain targeting.

Building upon the foundation of N2B drug delivery, nanotechnology has been increasingly explored as a means to enhance its efficiency and overcome formulation-related barriers.^8,17,18^ Nanoparticles (NP) can improve drug solubility, protect labile molecules from enzymatic degradation, and facilitate controlled release, while also enabling surface functionalization for targeted delivery.^19^ Among the various nanocarrier systems investigated, chitosan (CS)-based nanoparticles have garnered particular attention due to the favorable physicochemical and biological properties of this natural cationic polysaccharide. CS exhibits excellent mucoadhesiveness, biodegradability, biocompatibility, and the ability to transiently open tight junctions in the nasal epithelium, thereby enhancing paracellular transport of the therapeutic agent.^20^ Furthermore, CS can serve both as a matrix material and as a surface coating for hybrid nanostructures, extending its utility across a wide range of nanotechnological platforms.^21^

This review presents a comprehensive and critical analysis of the recent advances in the development of CS-based NPs for N2B delivery. We discuss their formulation strategies, mechanisms of action, and preclinical outcomes in diverse CNS pathologies, including neurodegenerative, neuropsychiatric, oncologic, and infectious diseases. We also integrate insights from recent patent filings, underscoring the translational potential and innovation landscape of these systems.

## 2 Central Nervous System Disorders

Disorders affecting the CNS represent a complex and heterogeneous group of conditions with profound implications for public health, quality of life, and healthcare systems worldwide.^22^ These include neurodegenerative diseases, neuropsychiatric disorders, brain tumors, and CNS infections—each with distinct etiologies, pathophysiologies, and clinical trajectories. Collectively, CNS disorders rank among the leading causes of disability and death, and their incidence continues to rise, driven largely by global population aging and the increasing prevalence of mental and neurological conditions.^23^

Despite this diversity, many CNS disorders share common hallmarks, such as progressive neuronal dysfunction, cognitive and behavioral impairments, and chronic disease progression.^24^ These features are often accompanied by complex symptom clusters, frequent comorbidities, and a high social and economic burden. Moreover, the clinical management of CNS disorders remains highly challenging due to diagnostic complexity, limited therapeutic efficacy, and, particularly, constraints in drug delivery. Most treatments rely on invasive administration routes, such as intravenous or intrathecal delivery, which are associated with increased risks and low patient acceptance. Additionally, even when oral administration is feasible, systemic side effects frequently occur, highlighting the need for new approaches to CNS-targeted drug delivery.^25^

Figure 1 summarizes the key aspects of CNS disorders and provides a framework for the subsequent discussion. In the following subsections, we outline major CNS disorders that have been the focus of translational research in drug delivery, including neurodegenerative and neuropsychiatric conditions, primary brain tumors, and CNS infections. Particular attention is given to their epidemiological relevance, clinical impact, and current therapeutic limitations, which collectively underscore the urgent need for more effective treatment strategies.

**Figure 1:**
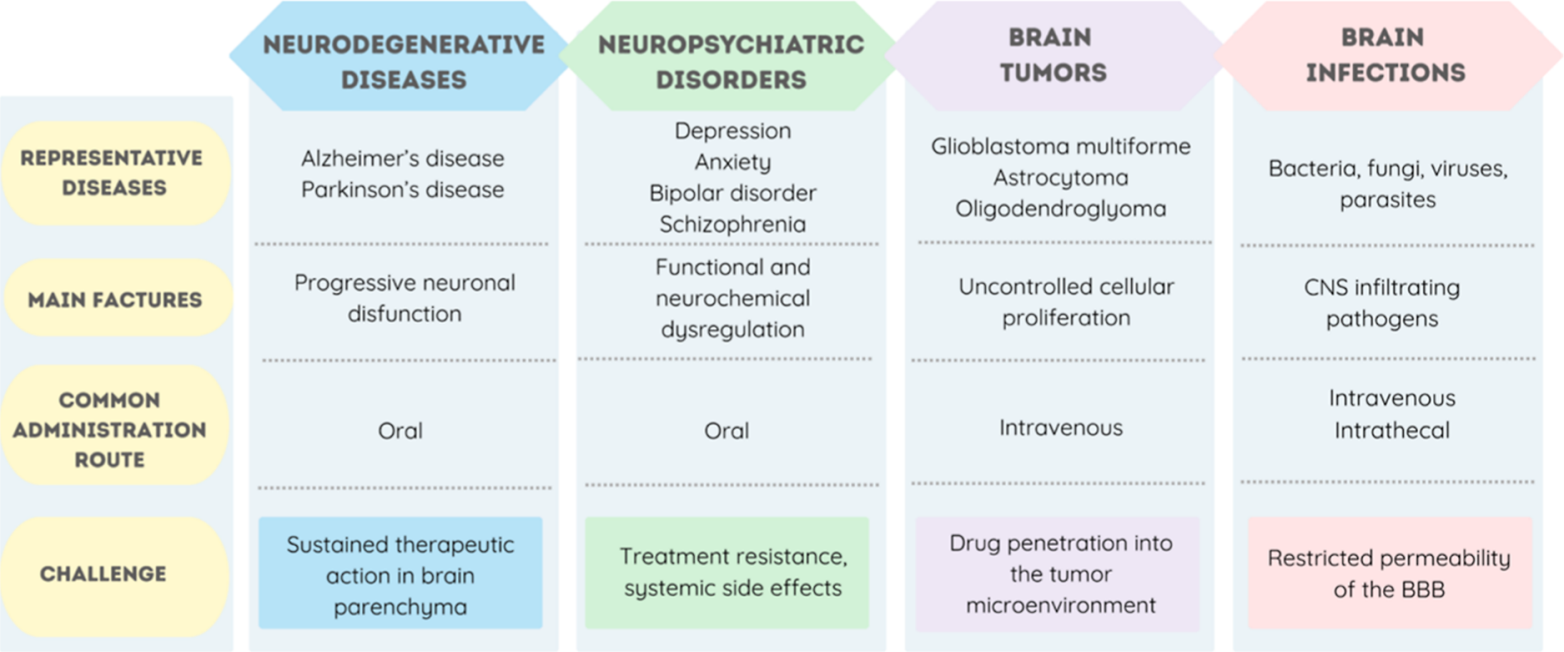
Schematic representation of key aspects of CNS disorders. Created using https://www.canva.com.

### 2.1 Neurodegenerative Diseases

Neurodegenerative diseases are among the most therapeutically intractable disorders of the CNS. Alzheimer’s disease (AD) and Parkinson’s disease (PD) are the two most prevalent forms, both characterized by progressive neuronal dysfunction and death, leading to irreversible cognitive, behavioral, and motor decline.^26,27^ AD currently affects approximately 51.6 million individuals worldwide, with projections reaching 132 million by the mid-21st century, alongside a 146.2% increase in mortality over the past decade.^28^ Similarly, the prevalence of PD has increased markedly, rising by approximately 156% between 1990 and 2019 and is projected to reach 22 million cases by 2050.^29^

Despite decades of research and partial elucidation of pathological hallmarks—such as amyloid-β plaques, hyperphosphorylated tau tangles in AD, and dopaminergic neurodegeneration in PD—clinically meaningful interventions remain limited.^30,31^ In AD, available pharmacological treatments offer only modest symptomatic relief without halting the disease progression. Most are administered systemically, including cholinesterase inhibitors (e.g., donepezil, rivastigmine) and *N*-methyl-d-aspartate (NMDA) receptor antagonists (e.g., memantine), which suffer from poor CNS penetration and frequent peripheral side effects.^32^ Recent advances in monoclonal antibodies targeting amyloid-β, such as lecanemab and donanemab, have shown limited efficacy and raised concerns regarding brain edema and infusion-related complications. Importantly, these biologics require intravenous delivery and long-term administration protocols, further complicating patient adherence.^33^

Similarly, PD management remains largely symptomatic. Levodopa remains the mainstay therapy, often combined with catechol-*O*-methyltransferase (COMT) inhibitors or dopamine agonists to prolong its effect.^31^ However, these agents do not modify disease progression and exhibit limited BBB permeability. Moreover, chronic use is associated with debilitating motor fluctuations and dyskinesias.^34^

Overall, the lack of targeted CNS-penetrant therapies underscores a critical need for innovative drug delivery strategies capable of overcoming the BBB and providing sustained therapeutic action within the brain parenchyma.

### 2.2 Neuropsychiatric Disorders

While neurodegenerative diseases are characterized by progressive neuronal dysfunction, neuropsychiatric disorders represent a distinct class of CNS conditions involving functional and neurochemical dysregulation. These disorders encompass a diverse spectrum of mental health conditions marked by intricate disturbances in mood, cognition, perception, and behavior, typically arising from dysregulation of CNS circuits.^35^ Among the most prevalent and debilitating disorders are major depressive disorder (MDD), anxiety disorders, BD, and schizophrenia (SCZ), each of which will be briefly examined in this review.

#### 2.2.1 Depression

MDD, commonly referred to as depression, is one of the most prevalent and disabling neuropsychiatric disorders, currently affecting more than 280 million people worldwide.^36,37^ Clinically, it manifests as persistent low mood, anhedonia (loss of interest or pleasure), fatigue, cognitive impairment, and pervasive feelings of guilt or worthlessness, often accompanied by disturbances in sleep and appetite. In severe cases, MDD can culminate in suicidal ideation or behavior.^38,39^

MDD is a heterogeneous, multifactorial condition, whose pathophysiology remains only partially understood. The long-standing and most widely accepted monoamine hypothesis posits that depressive symptoms arise from deficits in key neurotransmitters—particularly serotonin, norepinephrine, and dopamine.^37,38^ More recent evidence, however, implicates a broader array of mechanisms, including dysregulation of the hypothalamic–pituitary–adrenal axis; diminished neurotrophic support, exemplified by reduced brain-derived neurotrophic factor levels; chronic neuroinflammation marked by elevated pro-inflammatory cytokines such as interleukin-6 (IL-6) and tumor necrosis factor-alpha (TNF-α); hippocampal atrophy; and disturbances of circadian rhythmicity.^19,37^ In addition, both genetic susceptibility and environmental stressors are recognized as critical determinants of disease onset and progression.^40^

Despite significant advances in pharmacological interventions, MDD remains difficult to manage in a substantial proportion of patients. Conventional treatments primarily target monoaminergic pathways through agents such as selective serotonin reuptake inhibitors (SSRIs), serotonin–norepinephrine reuptake inhibitors (SNRIs), and monoamine oxidase inhibitors (MAOIs).^16^ However, nearly 50% of patients fail to achieve a satisfactory response to first-line therapy, and approximately 30% meet criteria for treatment-resistant depression.^31^ In addition to pharmacodynamic limitations, restricted drug delivery to the CNS, limited brain bioavailability, and interindividual variability in drug distribution further compromise therapeutic outcomes. Persistent residual symptoms, high relapse rates, and profound impairment in daily functioning underscore the urgent need for more effective and mechanistically targeted therapeutic strategies, including advanced drug delivery approaches.^34^

#### 2.2.2 Anxiety

Anxiety disorders represent one of the most prevalent categories of mental health conditions, affecting individuals of all ages worldwide. According to the World Health Organization, an estimated 359 million people worldwide, including approximately 58 million children and adolescents, currently live with an anxiety disorder.^39^ Their typically early onset, chronic course, high rates of comorbidity, and profound impact on daily functioning place anxiety disorders among the leading causes of health-related disability, accounting for approximately 3.3% of the global disease burden.^41^

Clinically, anxiety disorders comprise a broad spectrum of conditions, including generalized anxiety disorder, social anxiety disorder, panic disorder, separation anxiety disorder, specific phobias, agoraphobia, post-traumatic stress disorder, and obsessive-compulsive disorder. Although each exhibits distinct symptom profiles and age-related patterns of onset, these disorders often share overlapping neurobiological mechanisms and frequently co-occur.^42^

These disorders are typically marked by excessive and persistent fear, worry, or nervousness that can profoundly disrupt daily functioning, academic performance, occupational productivity, and interpersonal relationships.^43,44^ Beyond emotional distress, affected individuals often experience physical manifestations, such as tachycardia, hyperventilation, diaphoresis, gastrointestinal discomfort, and tremors. Importantly, anxiety disorders have also been associated with elevated cardiovascular morbidity and mortality, underscoring their considerable clinical and public health significance.^42^

The pathophysiology of anxiety is complex and has been incompletely elucidated. Neurobiological models implicate dysregulation of neural circuits governing fear processing and threat detection, particularly within the amygdala, hippocampus, and prefrontal cortex, as a central mechanism.^45^ At the molecular level, reduced γ-aminobutyric acid (GABA)–mediated inhibitory signaling contributes to heightened neuronal excitability and arousal. Moreover, alterations in serotonergic, noradrenergic, glutamatergic, endocannabinoid, and neuropeptidergic pathways have been linked to the development and persistence of anxiety symptoms.^46^

Pharmacological management of anxiety disorders has traditionally relied on benzodiazepines, which act on GABA-A receptors to produce rapid anxiolytic and sedative effects. However, concerns regarding dependence, tolerance, and cognitive adverse effects have prompted a therapeutic shift toward SSRIs, SNRIs, and nonbenzodiazepine anxiolytics such as buspirone, which are now widely regarded as first-line treatments.^44^

Despite these options, many patients fail to achieve full symptom remission: clinical trials report response rates of only 40–70%, with sustained remission occurring in merely 20–47% of the cases.^44^ Treatment-resistant anxiety remains a significant challenge, particularly among individuals with comorbid depression—a highly prevalent combination associated with greater symptom severity and poorer clinical outcomes.^47^ These limitations underscore the urgent need for more effective, accessible, and personalized therapeutic strategies.

#### 2.2.3 Bipolar Disorder

BD is a prevalent, heritable, and severely disabling psychiatric condition associated with a considerable economic burden. It is characterized by marked mood fluctuations, with alternating episodes of mania or hypomania and depression.^48,49^ BD is classified into two main subtypes: Bipolar I Disorder (BD I), which involves full manic episodes often accompanied by elevated mood, agitation, and increased energy, and Bipolar II Disorder (BD II), in which hypomanic episodes alternate with major depressive episodes.^50,51^ The global prevalence of BD is estimated to range from 1% to 5%, depending on the diagnostic subtype. While the incidence of BD I is similar in males and females, BD II appears to be more common in women. The disorder typically emerges in late adolescence or early adulthood, most often between the ages of 18 and 22.^49,52^ A major clinical concern in BD is its high rate of psychiatric comorbidities, including anxiety disorders, substance use disorders, personality disorders, and attention-deficit/hyperactivity disorder (ADHD).^53,54^

Although the pathophysiological mechanisms underlying BD are not yet fully understood, accumulating evidence implicates a complex interplay of genetic predispositions, environmental influences, neurodevelopmental dysregulation, neuroinflammatory processes, mitochondrial dysfunction, and disruptions in sleep and circadian rhythms.^55^ Pharmacological agents targeting various neurotransmitter systems—dopaminergic, serotonergic, glutamatergic, and GABAergic—have shown efficacy in both acute symptom management and long-term mood stabilization.^56^

The principal aim of pharmacological treatment in BD is the stabilization of mood to prevent the recurrence of acute episodes of mania, hypomania, and depression.^48,49^ Treatment regimens are typically individualized based on the patient’s symptom profile. Lithium, long considered the gold standard mood stabilizer, remains widely used and FDA-approved.^52^ Anticonvulsants such as carbamazepine and valproic acid, are particularly effective for manic and hypomanic episodes. Atypical antipsychotics, including haloperidol, olanzapine, quetiapine, and risperidone, are frequently utilized in the management of acute mood episodes.^57^ For depressive episodes, SSRIs and other antidepressants are often prescribed. Maintenance therapy commonly involves combination strategies, integrating agents such as lamotrigine, quetiapine, or olanzapine alongside lithium.^58^ Despite their clinical efficacy, these pharmacotherapies are associated with a range of adverse effects, including sedation, extrapyramidal symptoms, tremors, anxiety, metabolic disturbances, and weight gain, that may impair treatment adherence and necessitate close clinical monitoring.^49,52^

#### 2.2.4 Schizophrenia

Schizophrenia is a chronic and severely disabling psychiatric disorder affecting approximately 24 million individuals worldwide and ranking among the top ten causes of disability, owing to its broad range of functional impairments.^59,60^ Clinically, the disorder presents a spectrum of symptoms that are categorized as either positive or negative. Positive symptoms include hallucinations, delusions, and episodes of agitation or aggression. In contrast, negative symptoms encompass emotional withdrawal, social isolation, diminished spontaneity, and cognitive impairments.^61^

Although the precise etiology of schizophrenia remains unclear, current hypotheses suggest that its pathophysiology involves the dysregulation of several neurotransmitter systems. These include increased dopaminergic and serotonergic (5-HT) activity, alongside decreased glutamatergic activity—particularly via *N*-methyl-d-aspartate (NMDA) receptors and impaired gamma-aminobutyric acid (GABA) neurotransmission.^62^ Historically, schizophrenia was primarily linked to dopaminergic dysfunction, leading to the development of first-generation (typical) antipsychotics, such as chlorpromazine and haloperidol, which primarily target D2/D3 receptors.^63^ Second-generation (atypical) antipsychotics, including clozapine, risperidone, and quetiapine, exhibit broader receptor activity profiles.^64^

Despite their clinical utility, antipsychotic medications are associated with significant adverse effects, such as weight gain, extrapyramidal symptoms, hyperglycemia, sedation, and postural hypotension, that frequently compromise patient adherence to treatment.^61,63^ Furthermore, these drugs face pharmacokinetic limitations, including poor oral bioavailability, low aqueous solubility, and extensive first-pass metabolism.^65–67^ Treatment with antipsychotics often follows a cyclic pattern of remission and relapse. With each relapse, the duration required for symptom remission typically increases and therapeutic responsiveness diminishes. Consequently, early and effective intervention is critical to minimizing relapses and halting disease progression.^56^

### 2.3 Brain Tumors

In contrast to neuropsychiatric disorders, which primarily involve functional and neurochemical alterations, brain tumors are characterized by uncontrolled cellular proliferation, resulting in abnormal masses that disrupt normal neurological function. Without timely diagnosis and intervention, these neoplasms can cause profound neurological deficits and may ultimately lead to death.^68^ Histopathologically, brain tumors are classified into four grades based on their cellular characteristics and growth dynamics. Grades I and II are generally categorized as low-grade or slow-growing tumors, such as pilocytic astrocytoma and oligodendroglioma, and are typically associated with a more favorable prognosis. Conversely, grades III and IV represent high-grade or malignant tumors, including anaplastic astrocytoma and glioblastoma multiforme, which display marked aggressiveness, rapid progression, and dismal clinical outcomes.^69^

Glioblastoma multiforme (GBM) is the most common malignant subtype of gliomas and a primary intracranial neoplasm originating from glial cells. It is associated with an extremely poor prognosis, with approximately 30% of patients surviving up to one year and fewer than 5% beyond five years.^70^ Despite significant advances in research and therapeutic strategies, GBM remains associated with exceptionally high mortality rates and lacks effective treatment options capable of achieving a cure or substantially extending patient survival.^71^

GBM is marked by pronounced intratumoral heterogeneity, low immunogenicity, the presence of the BBB, and a marked resistance to conventional therapies. First-line treatment consists of maximal safe surgical resection followed by concomitant radiotherapy and adjuvant chemotherapy with Temozolomide (TMZ).^72^ One of the main obstacles to the development of effective therapeutic strategies is the BBB, whose selective permeability greatly restricts drug penetration into the tumor microenvironment. GBM cells further complicate this scenario by overexpressing vascular endothelial growth factor, which drives the formation of abnormal, highly permeable, and structurally deficient blood vessels.^71^ This vascular dysfunction disrupts tight junctions and compromises BBB integrity, paradoxically increasing permeability while hindering effective and uniform drug distribution.^73^

Nanotechnology and related drug-delivery technologies are being extensively explored to optimize drug bioavailability within the CNS before the initiation of therapeutic protocols for tumor treatment. Employing alternative routes of administration offers a promising strategy to bypass the BBB, thereby enabling more efficient and targeted delivery of therapeutics to the brain.^74^

### 2.4 Brain Infections

In addition to noninfectious conditions, infectious diseases of CNS represent a clinically significant and heterogeneous group of disorders, often associated with high morbidity and mortality and further complicated by challenges in effective drug delivery. These infections are caused by a diverse range of pathogens, including bacteria, viruses, fungi, and parasites, which further complicate therapeutic management.^75^ CNS-infiltrating pathogens exhibit tropism for specific brain cells including neurons and glial cells. Notably, viruses such as rabies virus, Zika virus, tick-borne encephalitis virus, and herpes viruses demonstrate a particular affinity for neurons.^76^ These infections frequently result in profound disruptions of CNS homeostasis, leading to severe neurological conditions such as encephalitis or meningitis, which may culminate in significant morbidity, long-term disability, or death.^77^ Other pathogens are more prone to invade the CNS under conditions of compromised physiological barriers or immune function, including immunodeficiency, systemic inflammation, BBB disruption, traumatic injury, neurosurgical interventions, or concurrent infections. Examples of such opportunistic pathogens include fungal species such as *Candida* spp., *Cryptococcus* spp., and members of the order *Mucorales*, the protozoan parasite *Toxoplasma gondii*, and bacterial species such as *Streptococcus pneumoniae*, *Staphylococcus aureus*, and *Mycoplasma* spp.^78^

The treatment of brain infections remains a formidable clinical challenge. Although several pharmacological agents are currently available, their therapeutic effectiveness is often compromised by the restricted permeability of the BBB, which prevents most conventional drugs from achieving therapeutic concentrations within the CNS.^79^ As a result, only a limited number of therapeutic options exist for CNS and brain infections, and those currently in clinical use frequently exhibit suboptimal efficacy in pathogen eradication. To address these limitations, recent research has focused on the development of innovative therapeutic approaches, particularly nanotechnology-based formulations and advanced drug delivery systems designed to enhance BBB penetration and improve drug bioavailability at the target site.^80^

## 3 Nose-to-Brain Delivery

Delivery of drugs to the CNS poses multiple challenges. Many compounds that are effective against diseases of this system cannot cross the BBB, frequently displaying low solubility and poor central bioavailability.^81^ The BBB separates the cerebral capillary blood from the brain’s interstitial fluid through a structure formed by endothelial cells, astrocytes, and pericytes. Under physiological conditions, only lipophilic and low-molecular-weight substances can passively diffuse across the BBB. However, this transport is limited by the physicochemical properties of the drug, and often requires specific mechanisms such as paracellular transport, ion channels, ligand-specific receptors or carriers, and energy-dependent transport systems.^82^

Several strategies have been employed for the treatment of CNS diseases, including intrathecal and intracerebroventricular injections, as well as conventional routes such as oral and intravenous administration.^83^ While invasive routes can achieve higher CNS drug concentrations, they are associated with procedural complexity, increased risks, and restriction to hospital settings. In contrast, conventional delivery routes are limited by the BBB, resulting in poor CNS bioavailability and limited brain targeting. Consequently, high systemic doses are often required, increasing the risk of peripheral toxicity, systemic side effects, gastrointestinal disturbances, and extensive hepatic metabolism.^84,85^

In this context, N2B delivery has emerged as a promising strategy for directly transporting drugs to the brain through the olfactory and trigeminal nerve pathways, thereby bypassing the BBB, minimizing systemic side effects, and enabling targeted delivery to the CNS.^11^ The human nasal cavity has an approximate surface area of 160 cm^2^, and drug transport occurs across both the respiratory and olfactory epithelia, with the olfactory region serving as the primary pathway.^12,13^ The respiratory region, which constitutes the largest portion of the nasal cavity, is lined with a ciliated respiratory epithelium and functions primarily as a protective surface. It is innervated by branches of the trigeminal nerve. In contrast, the olfactory region, which represents about 10% of the nasal cavity surface area, is located in the upper part of the cavity and is innervated by fine fibers originating from the olfactory nerve.^86^ Both regions, as well as their respective neural connections, are depicted in Figure 2.

**Figure 2:**
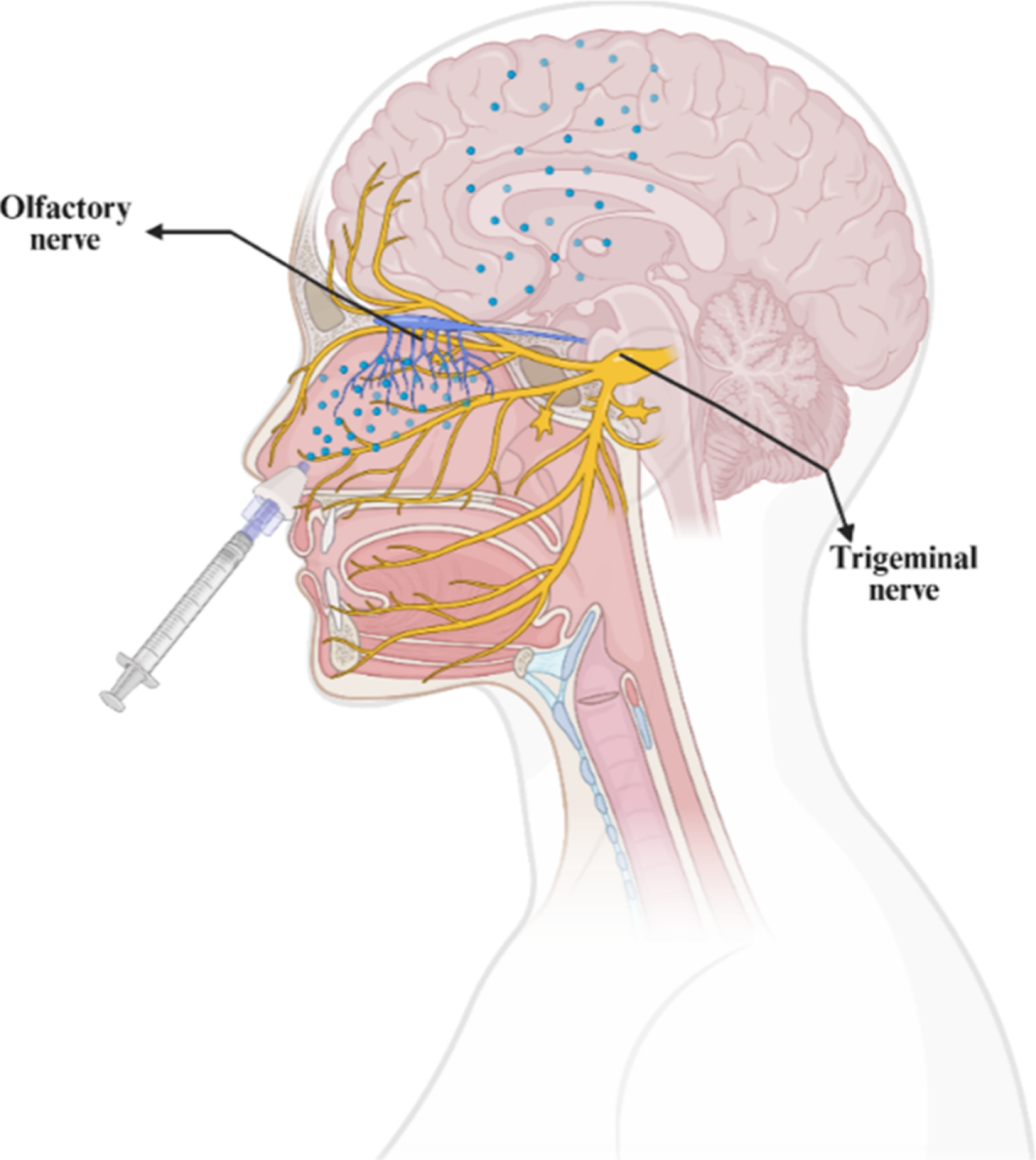
Schematic illustration of the nose-to-brain delivery pathway via the olfactory and trigeminal nerves. Created using https://www.biorender.com/.

Drug transport along these pathways can occur through the olfactory epithelium either by rapid extracellular movement via perineural and perivascular spaces or by slower intra-axonal transport along olfactory neurons, ultimately reaching the olfactory bulb and higher brain regions.^87^ Olfactory nerve fibers cross the cribriform plate of the ethmoid bone and project to the olfactory bulb within the CNS. In contrast, the trigeminal nerve pathway involves the ophthalmic and maxillary branches, which extend to both the olfactory and respiratory epithelia.^88^ Drugs may travel along these nerves primarily through perineural routes and, to a lesser extent, by intra-axonal transport, reaching the CNS at the level of the pons and terminating in the spinal trigeminal nucleus within the brainstem.^89^ Notably, the transit time along the trigeminal nerve has been reported to exceed that of the olfactory route by more than 10 h.^90^

Compared with other administration routes, such as parenteral and intrathecal, the intranasal route is considered noninvasive, easy to self-administer, and painless.^91^ Nevertheless, several formulation parameters must be carefully evaluated during the development of the nasal dosage forms. Among these, pH is particularly important and should be maintained between 4.5 and 6.5 to prevent irritation of the nasal mucosa, inhibit the growth of pathogenic microorganisms, and preserve normal ciliary function.^92^ Another critical parameter to consider is the volume of administration, as excessive volumes can cause mucosal irritation and may result in anterior leakage from the nostrils or posterior drainage into the pharynx, leading to patient discomfort. The optimal volume for intranasal administration typically ranges from 0.05 to 0.15 mL, with a recommended maximum of 0.20 mL.^88^ To enhance therapeutic efficacy, particularly through the olfactory and trigeminal pathways, specialized nasal drug-delivery devices have been developed, including droppers, syringes, pressurized metered-dose inhalers, breath-powered bidirectional nasal devices, and pressurized olfactory delivery systems.^93^

To date, several intranasal drug delivery formulations have been approved for clinical use, and there is growing interest in repurposing and developing N2B delivery systems to enhance therapeutic outcomes in CNS disorders.^13^ These products target a range of clinical conditions, including multifactorial disorders that involve both the peripheral and central nervous systems, such as migraine, opioid overdose, and dry eye disease, as well as neurological and psychiatric conditions that are primarily associated with CNS dysfunction, such as epilepsy and depression. The approved intranasal formulations currently available on the market are summarized in Table 1.

**Table 1:** FDA-Approved Nose-to-Brain Formulations^a^

active ingredient	therapy	approval time/Country	device
Nicotine	Smoking cessation	1996/USA	Reusable spray device
Sumatriptan	Migraine	1997/UK	Disposable prefilled nasal spray device
Dihydroergotamine mesylate	Migraine	1997/Canada	Nasal spray device
Zolmitriptan	Migraine	2003/USA	Disposable prefilled nasal spray device
Nalocone hydrochloride	Opioid overdose	2015/Ireland	Disposable prefilled nasal spray device
Sumatriptan	Migraine	2016/UK	Xsail system
Sumatritpan	Migraine	2019/USA	Disposable prefilled nasal spray device
Midazolam	Epilepsy	2019/Belgium	Disposable prefilled nasal spray device
Esketamine hydrochloride	Depression	2019/USA	Disposable prefilled nasal spray device
Diazepam	Epilepsy	2020/USA	Disposable prefilled nasal spray device
Dihydroergotamine mesylate	Migraine	2021/USA	POD system
Nalozone hydrochloride	Opioid overdose	2021/USA	Disposable prefilled nasal spray device
Varenicline	Dry Eye Disease	2021/USA	Reusable nasal spray device
Zavegepant	Migraine	2023/USA	Disposable prefilled nasal spray device
Naloxone hydrochloride	Opioid overdose	2023/USA	Disposable prefilled nasal spray device

a Adapted with permission from [Ge et al.][Bibr ref13] Copyright [2024] MDPI.

In addition to currently approved therapies, several clinical studies investigating N2B delivery have been completed or are ongoing, highlighting the translational potential of this approach. A completed clinical trial evaluating intranasal Sumatriptan administered via a dedicated delivery device demonstrated a faster onset of action compared to oral administration, with significant reductions in migraine pain intensity and migraine-related disability observed as early as 10 min postdose.^94,95^ Additionally, a completed study assessing intranasal reduced glutathione (GSH) showed that this approach effectively increases brain GSH levels, with elevated concentrations persisting for at least 1 h in patients with PD.^96,97^

Intranasal insulin has also been explored in clinical trials, demonstrating safety and clinically relevant improvements in cognitive and functional performance in patients with PD following daily administration over 4 weeks.^98,99^ Furthermore, a completed study investigated its effects over a 12 week treatment period, revealing its ability to modulate cognitive outcomes, as well as blood and cerebrospinal fluid biomarkers and amyloid-β deposition in patients with AD.^100,101^ In addition to these findings, an ongoing clinical trial is recruiting participants to evaluate the effects of intranasal oxytocin on stress, anxiety, and depression in caregivers of individuals with dementia.^102^

However, nasal drug delivery also faces anatomical and physiological challenges, such as interindividual variations in nasal cavity structure, significant differences between human and animal models (making this difficult to extrapolate preclinical studies to clinical) and the influence of mucociliary clearance.^14^ This last one is an interaction between the cilia and mucus layers, which helps inhaled toxic substances to adhere and transport toward the nasopharynx and gastrointestinal tract. The average clearance rate is approximately 6 mm/min and this rapid turnover can significantly impact drug bioavailability in nose-to-brain delivery, as the formulation must remain in contact with nasal epithelium long enough to penetrate the mucus and adhere to the local nasal epithelium before being washed away.^19^

To address the inherent limitations of N2B drug delivery, various nanoparticulate systems have been developed to enhance drug stability within the nasal cavity, facilitate *trans*-epithelial transport, and improve drug targeting to the CNS.^103^ Nanoparticles with sizes ranging from 100 to 200 nm are considered optimal for N2B delivery, since this size range facilitates cellular uptake and enhances the ability of the particles to penetrate the nasal mucus, enabling drug transport through the olfactory and trigeminal nerves to the brain.^15,16,104^ In addition, nanoparticles can assist in the precise control of drug release kinetics, which may help maintain therapeutic concentrations in the brain, potentially enhance treatment efficacy, and reduce dosing frequency.^105^ Moreover, their biocompatibility and low toxicity can help minimize potential adverse effects on both nasal and cerebral tissues.^106^

Building on these advantages, several preclinical studies have demonstrated the translational potential of nanoparticle-based systems for N2B delivery. For instance, intranasal lipid nanocapsules loaded with nimodipine achieved brain drug levels comparable to those obtained by intravenous administration while significantly reducing systemic exposure—an important advantage given the cardiovascular side effects associated with intravenous delivery. This reduced peripheral distribution, combined with sustained brain availability, highlights the potential of nanocarriers to improve safety and therapeutic efficacy.^107^ Similarly, in an induced rat model of parkinsonism, carbenoxolone-loaded CS-coated solid lipid nanoparticles showed superior neuroprotective effects compared to a drug suspension, preserving neuronal architecture and attenuating oxidative stress and apoptosis.^15^

Importantly, this translational potential is further supported by ongoing clinical investigations, including a phase II trial evaluating an intranasal nanoparticle formulation (APH-1105) in patients with mild to moderate AD, reinforcing the clinical relevance of nanotechnology-based N2B strategies.^108^

A wide variety of nanocarrier platforms—including liposomes, polymeric nanoparticles, solid lipid nanoparticles, and dendrimers—provide versatile strategies for drug encapsulation and enable the tailoring of delivery profiles.^109^ Among these, CS-based nanoparticles stand out as a particularly promising system owing to their strong mucoadhesive properties, which are discussed in the following section.

## 4 Chitosan-Based Nanoparticles for N2B Delivery

Chitosan (CS) is a linear cationic polysaccharide composed of β-(1 → 4)-linked d-glucosamine and *N*-acetyl-d-glucosamine units, obtained through the partial or complete deacetylation of chitin, a naturally abundant biopolymer primarily found in crustacean shells and fungal cell walls.^20,110^ Each repeating unit of CS contains two hydroxyl groups and one amino group, as illustrated in Figure 3. CS is classified as a weak base, remaining insoluble in water and most organic solvents. However, it becomes soluble in dilute acidic environments (pH < 6.5), where protonation of the amino groups (R–NH_2_ → R–NH_3_^+^) confers a positive charge and enhances solubility. Under neutral or alkaline conditions, deprotonation occurs, resulting in precipitation driven by charge loss and strengthened intermolecular interactions.^21,111^

**Figure 3:**
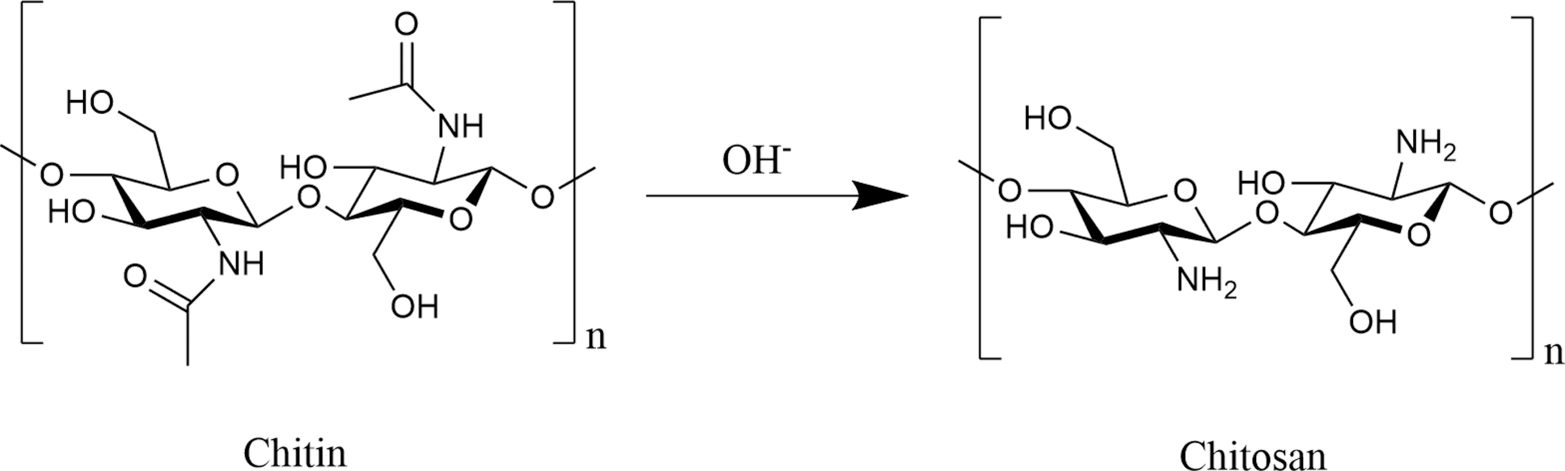
Schematic representation of chitin deacetylation leading to the formation of CS. Created using ChemDraw Professional 15.0.

Beyond its structural characteristics, CS possesses a set of properties that make it an attractive candidate for biomedical and pharmaceutical applications. These include excellent biocompatibility, biodegradability, and low toxicity, together with a wide spectrum of biological activities such as antimicrobial, anti-inflammatory, anticancer, and antioxidant effects.^112–116^ Owing to its cationic nature, CS readily engages in electrostatic interactions with negatively charged biological membranes and mucosal surfaces, thereby prolonging residence time and improving drug absorption and bioavailability.^20,117^ Such mucoadhesive properties are particularly advantageous for N2B delivery, where mucociliary clearance constitutes a major barrier. In addition, CS can transiently modulate epithelial tight junctions through interactions with proteins such as occludin and zonula occludens-1 (ZO-1), thereby enhancing paracellular drug transport across the nasal epithelium.^19,117^ Taken together, these features highlight the potential of CS as a foundational material for developing advanced drug delivery systems, particularly nanoengineered platforms tailored to overcome the barriers inherent to N2B administration.

Beyond these biological and physicochemical attributes, the versatility of CS also arises from its functionalizable backbone, which enables chemical modifications to improve solubility, enhance drug loading within nanoparticulate systems, and increase targeting specificity.^19,21,116^ In this context, several studies have investigated CS derivatives bearing chemical modifications that expand their functional versatility. Examples include quaternary ammonium palmitoyl glycol chitosan (GCPQ) (Figure 4A), which enhances drug transport across the gastrointestinal epithelium, BBB, and cornea;^118–120^ carboxymethyl chitosan (CMCh);^121^ and *N*,*O*-carboxymethyl chitosan (N,O-CMCS) (Figure 4B), which improves aqueous solubility, mucoadhesion, and biocompatibility, thereby facilitating nanoparticle stability and effective permeation through the nasal mucosa.^122,123^ Taken together, these features position CS as a promising polymeric platform for engineering advanced nanosystems designed to overcome the barriers of N2B administration.

**Figure 4:**
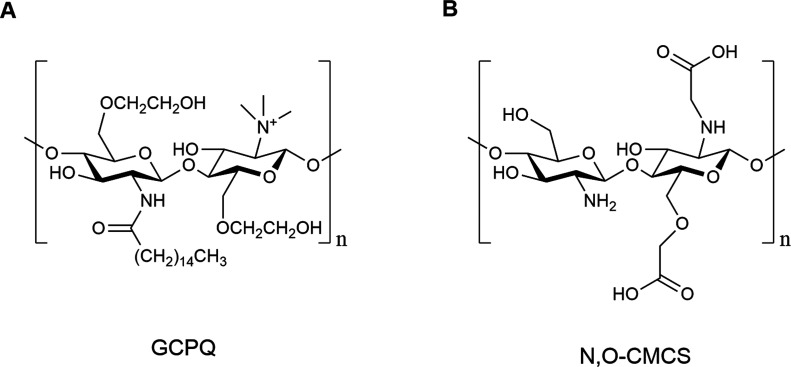
Schematic representation of CS derivatives. (A) quaternary ammonium palmitoyl glycol chitosan (GCPQ); (B) N,O-carboxymethyl chitosan (N,O-CMCS). Created using ChemDraw Professional 15.0.

CS-based nanoparticles can be produced using several established techniques, including ionic gelation,^124^ reverse micelles (microemulsion),^37^ emulsification,^125^ coacervation,^126^ nanoprecipitation,^127^ and spray-drying.^128^ The choice of method depends on factors such as the physicochemical properties of the encapsulated drug, desired particle size, formulation safety, and scalability.^117,129^ Among these approaches, ionic gelation remains the most widely employed owing to its simplicity, aqueous processing, and avoidance of toxic cross-linkers or solvents, making it a cost-effective, scalable, and environmentally sustainable method.^20,21,130,131^

In addition to functioning as a matrix material, CS can also be employed as a surface coating for a wide range of nanostructures, including polymeric nanoparticles,^132^ solid lipid nanoparticles,^105,133,134^ and liposomes.^135,136^ The CS coating can be applied either during nanoparticle formation or postassembly by introducing a CS solution under controlled mixing conditions. The coating process primarily occurs through polymer chain entanglement and/or electrostatic interactions between the positively charged amino groups of CS and the negatively charged functional groups present on the surface of lipid or polymeric nanoparticles.^21^ This approach confers several advantageous properties to the delivery system, including enhanced mucoadhesiveness, improved permeability, greater colloidal stability, and augmented biological activity,^20^ thereby reinforcing the role of CS as a versatile component in nanoparticle engineering.

In the specific context of N2B administration, CS-based nanotechnology has gained increasing attention as a versatile strategy for CNS drug delivery. Formulations employing CS-based or CS-coated nanoparticles are being explored across a wide range of indications—including neurodegenerative,^15,16^ neuropsychiatric,^121^ oncological,^104^ and infectious CNS^128^ disorders—underscoring their translational potential as alternatives or complements to conventional therapies.

The physicochemical characteristics of CS-based nanoparticles, such as particle size and surface charge, are critical for efficient N2B delivery. Smaller nanoparticles favor direct brain uptake through the nasal route, while sizes within 100–200 nm are generally considered optimal; however, larger CS-based systems (>200 nm) have also shown effective brain transport.^137,138^ A zeta potential exceeding ±20 mV is essential to ensuring sufficient electrostatic repulsion between particles, thus preventing aggregation and enhancing colloidal stability. Moreover, the typically high and positive surface charge of CS nanoparticles promotes adhesion to the negatively charged nasal mucosa, increasing residence time and facilitating cellular transport.^139,140^ Altogether, these parameters strongly influence the therapeutic performance of CS nanocarriers during intranasal administration. In addition to these physicochemical characteristics, the interaction of CS nanoparticles with nasal epithelial cells plays a crucial role in determining their internalization pathways.^141^

Although the underlying mechanisms are not yet fully elucidated, several studies suggest that CS nanoparticles enhance paracellular transport by transiently modulating tight junction integrity through electrostatic interactions with negatively charged junctional proteins, including claudins, occludin, and ZO-1, thereby facilitating drug permeation without compromising epithelial integrity. Cellular uptake of these nanoparticles occurs primarily via clathrin- and caveolae-mediated endocytosis, while macropinocytosis contributes as a secondary internalization pathway.^141,142^ Formulation parameters play a critical role in delivery efficiency as controlled increases in viscosity can enhance deposition in the olfactory region, thereby improving brain targeting. Moreover, stimuli-responsive systems enable site-specific drug release within diseased brain regions, via intraneuronal transport along axons or extraneuronal diffusion through perineural pathways.^143,144^

This section provides a comprehensive and critical review of the current literature on CS-NP for N2B delivery with emphasis on their capacity to enhance drug bioavailability, improve therapeutic outcomes, and address unmet needs in CNS treatment.

### 4.1 Therapeutic Applications and Current Evidence

Several drugs have been encapsulated in CS-based nanoparticles to enhance their pharmacological efficacy in the treatment of CNS disorders, as summarized in Table 2. These formulations have been developed using different strategies, including CS-coated nanoparticles,^15,32,105,134,145–147^ CS-based polymeric nanoparticles,^148–151^ polymer blends incorporating CS,^137,141,147,152^ and hybrid systems.^67,132,138,148,153–155^ In addition, modified preparation approaches employing cyclodextrins as cross-linking agents have also been reported.^156^

**Table 2:** CS-Based Nanoparticles for Nose-to-Brain Delivery in CNS Disorders^a^

neurodegenerative disorders – Alzheimer’s disease (AD)				
drug	carrier	limitations	main results	ref
Narigenin	CS-NP	Low aqueous solubility, low bioavailability	Memory improvement, higher levels of endogenous antioxidants, higher neuronal density	^16^
Sinapic Acid	CS-coated-NLC	Low aqueous solubility and low permeability through BBB	Improved nasal permeation, increased half-life, and brain concentration after intranasal administration	^106^
Rivastigmine	CS-NP	Short half-life, lower bioavailability, and lower brain concentration after oral administration	Downregulation of caspase-3 and lower expression of Tau after intranasal administration	^167^
Donezepil	CS-NP	Low ability to cross BBB	Higher brain concentration after intranasal administration	^160^
Quercetin	CS-coated fullerene conjugate	Low bioavailability, solubility, and rapid metabolism	Improvement of mucoadhesion and of nasal permeation in	^163^
17β-estradiol	CS-coated-NLC	Peripheral side effects and risk of breast cancer	Improvement of learning ability and long-term memory	^140^
Vinpocentine	In situ gel based on CS-NP	Low oral bioavailability	Improved nose-to brain distribution higher brain concentration after nasal administration	^164^
Lutein	CS-NP	Low solubility, lower bioavailability	Cellular uptake by the caveolae-mediated endocytosis, clathrin-mediated pathway, and micropinocytosis. Higher penetration into BBB model, higher brain distribution	^142^
Berberin	CS-coated-NLC	Poor systemic bioavailability, limited CNS penetration	Improved nasal permeation and brain concentration, Highest nose-to brain-distribution	^165^
Resveratrol	SPIONs-loaded CS coated bilosomes	Poor bioavailability, low aqueous solubility, photodegradation, and extensive metabolization	Improved in cognitive and memory functions	^154^
Insulin	CS-coated-SLN	Impaired transport across BBB	Higher nasal permeability to coated-SLN	^161^
Ferulic Acid	CS-coated-SLN	Poor aqueous solubility, low permeability across lipophilic barriers, and extensive first-pass metabolism	Improved drug permeation through nasal mucosa, higher brain distribution, improvement in cognitive ability	^159^
Meloxicam (MEL)	CS-coated-SLN	Lipophilicity, low brain bioavailability	Improved mucoadhesion and nasal permeation to CS coated NP regarding uncoated, higher permeation to MEL-SLN than MEL-PLGA NP	^32^
Galantamine	CS-NP	Adverse effects related to oral administration	Reduction of amyloid-β deposition, suppression of Notch signaling an, improvement of brain delivery and of half-life time, higher nose-to-brain transport	^149^
Curcumin	Core–shell NP based on CS shell and PLGA as the core	Low solubility, low bioavailability, and extensive first-pass metabolism	Improved nasal mucosa permeation, cellular uptake by calveolae-mediated endocytosis, higher in vitro BBB permeation and brain distribution, Cellular uptake by caveolae-mediated endocytosis, clathrin-mediated pathway and micropinocytosis	^141^
Huperzine A	PLGA NP with surface modification by Lf-conjugated TMC	Lack of brain selectivity, serious gastrointestinal side effects	Higher adsorption to mucin regarding PLGA-NP and TMC-NP, higher brain fluorescence and higher targeting-efficiency to memory-related areas	^162^
BACE1 siRNA	CS-coated-SLN	Short half-life	Improved permeation into caco-2 cells	^133^

Neurodegenerative disorders—Parkinson’s disease (PD)				
drug	carrier	limitations	main results	ref
Carbenoxolone	CS-coated-SLN	Poor aqueous solubility, steroid-like structure	Improvement in motor functions, coordination, higher levels of dopamine in brain	^15^
Rotigotine	Lecithin-CS-NP	Poor oral bioavailability, low aqueous solubility, and extensive first-pass metabolism	Higher nasal permeation and nasal mucociliary transport, higher brain concentration	^153^
Levodopa	GCPQ-NP	Low oral bioavailability, limited brain uptake, peripheral side effects, and poor brain bioavailability	Higher dopamine levels in the brain after nasal administration	^120^
Dopamine	N,O-CMCS-NP	Poor solubility and/or unstable the gastrointestinal fluids	Higher Olfactory Ensheathing Cells uptake due to mucin-N,O-CMCS-NP complex	^123^
Piribedil	Thermoresponsive gels containing Lecithin-CS-NP	Low oral bioavailability, gastrointestinal side-effects	Increased of the relative bioavailability of PBD in brain of rats, higher nose-to brain distribution, and higher targeting efficiency	^138^
Ropinirole hydrochloride	PLGA/CS-NP	Low half-life time, extensive hepatic first-pass metabolism, low bioavailability	Higher nasal mucosa permeation to rospirenone-PLGA/CS-NPs than rospirenone loaded-PLGA/NP	^152^
Rotigotine	CS-NP	Low aqueous solubility, extensive first-pass metabolism, and low bioavailability	Reduced catalepsy, akinesia and total immobility time enhanced nose-to-brain transport, higher brain drug targeting efficiency	^148^
Narigenin	CS-NP	Gastrointestinal degradation, inefficient permeability, low aqueous solubility, low bioavailability	Higher permeation through nasal mucosa, reduced oxygen reactive species due to narigenin-antioxidant effect uptake	^150^
Pramipexol	CS-NP	Low brain bioavailability	Reduction of catalepsy score, improvement in cerebral dopamine, glutathione, and catalase levels	^139^
Rasagiline	CS-coated PLGA NP	Inability to reach high concentration in brain tissue and low oral bioavailability	Higher nasal mucosa permeation and enhanced brain-to-nose distribution	^157^
Rasagiline	CS-glutamate NP	Short half-life and low oral bioavailability due to hepatic first pass effect, gastrointestinal adverse effects	Higher nasal mucosa permeation, higher brain concentration, enhanced nose-to brain distribution, higher drug targeting efficiency	^158^
Dopamine	Glycol-CS/sulfobutylether-β-cyclodextrin NP	Inability to overcome the BBB, extensive metabolism by liver when orally administered	Repeated intranasal administration improved dopamine levels in the right nostril of rats	^156^
Rotigotine	Lecithin-CS-NP	Poor oral bioavailability, low aqueous solubility, and extensive first-pass metabolism	Higher nasal permeation and nasal mucociliary transport, higher brain concentration	^153^

neuropsychiatric disorders—anxiety				
drug	carrier	limitations	main results	ref
Buspirone	CS-coated-NLC	Low oral bioavailability	Higher brain drug targeting efficiency, highernose-to-brain transport	^134^
Riluzole	Transferin/CS-NP	Low oral bioavailability	Enhanced brain uptake, higher brain concentration after intranasal administration, better anxiety effect, increase in glutathione levels, reduction of malondialdehyde levels	^124^
Buspirone hydrochloride	ThiolatedCS-NP	Low oral bioavailability	Higher brain concentrations after intravenous and intranasal administration, improved nasal mucosa adhesion, higher nose-to-brain transport, lower anxiety effect—i.n. administration of TCS resulted in higher brain concentrations than BUH solution administered either i.v. or i.n.	^145^

neuropsychiatric disorders—bipolar disorder				
drug	carrier	limitations	main results	ref
Lithium	Hydrogel based on functionalized oxidized starch NP	Low therapeutic index	Reduction of hyperlocomotion and lower plasma concentrations after intranasal administration	^121^

neuropsychiatric disorders—depression				
drug	carrier	limitations	main results	ref
Paroxetine hydrochloride	CS-coated-PLGA-NP	Low bioavailability	Higher brain concentration after nasal administration, reduced depression effects	^132^
Mirtazapine	CS-grafted cationic leciplexes	Low bioavailability	Reduction of depression, higher brain concentration higher brain relative bioavailability after nasal administration	^171^
Imipramine hydrochloride	CS-NP embedded in in situ thermoresponsive gel	Low bioavailability and gastrointestinal side-effects	Improved nasal mucosa permeation regarding nongel CS-NP	^172^
Duloxetine hydrochloride	CS-grafted-PLGA/PVA-NP	Low bioavailability	Lower depression effects, higher brain relative bioavailability after nasal administration	^125^
Desvenlafaxin succinate	PLGA/CS NP	Inability to reach brain after oral administration	Enhanced nose-to-brain transport, Reduced depression symptoms	^147^
Selegiline hydrochloride	CS-NP	Low bioavailability	Higher brain concentration, reduced oxidative stress, reduction of immobility	^155^

Neuropsychiatric disorders—Schizophrenia				
drug	carrier	limitations	main results	ref
Lurasidone hydrochloride	Transferrin conjugated CS-NP	Short-half-life, low oral bioavailability, high drug dosing, and drug-related toxicity	Improvement in locomotion, reduced catalepsy, activity; higher brain targeting-efficiency, higher nose-to-brain transport	^173^
Ziprazidone Hydrochloride	Transferrin conjugated CS-NP	Low bioavailability, poor solubility, and extensive first-pass metabolism	Higher nasal permeation reaching deeper mucosa layers, absence of harmful effects on nasal mucosa	^174^
Quetiapine	Poloxamer-CS	Limited bioavailability, hematological side effects	Higher cell uptake, higher nasal mucosa permeation	^65^
Lurasidone hydrochloride	CS-NP	Low bioavailability	Reduction of catalepsy and higher locomotion, higher brain concentration after nasal administration	^175^
Peptide drug	Hydrogel based on starch NP/O-carboxymethyl CS	Highly degraded by digestive enzyme by oral administration	Reduction of schizophrenia negative symptoms	^176^
Asenapine maleate	CS-coated-mucoadhesive nanoemulsion	Biopharmaceutics Classification System class II	Improved brain concentration after intranasal administration, reduction of extrapyramidal effects	^177^
Risperidone	CS-NP	High hydrophobicity, extensive hepatic metabolism, and varied bioavailability	Higher plasma half-life after nasal administration, improvement in antipsychotic effect	^178^
Risperidone	CS-coated-lipid nanoparticle	Low aqueous solubility, low bioavailability, and high protein binding	Reduction of catalepsy and increased locomotion, higher brain targeting-efficiency	^67^
Olanzapine	CS-coated niosomes	Low aqueous solubility, low bioavailability	Higher nasal permeation and brain concentration after intranasal administration	^66^
Asenapine maleate	Glycol chitosan (GC)- coated-NLC	Low bioavailability, embryonary, and reproductive adverse effects	Higher mucoadhesion in nasal mucosa, higher brain absolute bioavailability, higher brain targeting-efficiency, no teratogenic effect	^179^
Olanzapine	CS-NP	Low bioavailability, Extensive first pass metabolism	Lower cytotoxicity than olanzapine solution, absence of harmful effects to nasal mucosa	^180^

Brain tumors				
drug	carrier	limitations	main results	ref
Gemcitabine	CS-coated-PLGA-NP	Side effects, extensive metabolization	Improved mucoadhesion, higher ability to inhibit cell survival	^104^
Isovanillin	CS-NP	Chemical instability	Improved nasal permeability and higher cytotoxicity, highest brain concentration after nasal administration	^168^
Simvastatin	CS-coated-loaded lipid-core nanocapsules	Limited ability to cross BBB	Decreased tumor size, higher cytotoxicity, and higher brain concentration after intranasal administration	^105^
Alpha-cyano-4- hydroxycinnamic acid and cetuximab	PLGA/CS NP	Chemical instability	Higher nasal permeation and tumor size reduction	^137^
Alpha-cyano-4-hydroxycinnamic acid	CS-loaded PLGA/OCS NP	Extensive first-pass metabolism	Improvement of cytotoxic and antiangiogenic effects	^74^
Kaempferol	CS-coated-nanoemulsions	Prone to oxidation	Higher mucoadhesion, decreased viability of glioma cells	^146^
Anti GaL-1 siRNA	CS-NP	Low short-life	Reduction of GaL expression, distribution from nose-to-brain, ability to cross BBB model	^151^

brain infections				
drug	carrier	limitations	main results	ref
Efavirenz	CS-*g*-hydroxypropyl β-cyclodextrin nanoparticles	Biopharmaceutics Classification System class II	Higher permeability in nasal mucosa, Higher nose-to-brain distribution regarding efavirenz solution	^169^
Artemether and lumefantrine	Trimethyl CS coating of lipidic nanocarriers	Poor aqueous solubility of drugs	Higher nasal mucosa permeability and antiplasmodial effect	^170^
Isoniazid and rifampicin	Spray-dried CS-NP	Poor ability to cross BBB	Bactericidal effect in mice, higher brain distribution to spray-dried rifampicin loaded, sustained release of spray-dried isoniazid-loaded CS NP	^128^

a BBB: blood–brain barrier; CMCh: carboxymethyl chitosan; CS: Chitosan; GCPQ: *N*-palmitoyl-*N*-monomethyl-*N*,*N*-dimethyl-*N*,*N*,*N*-trimethyl-6-*O*-glycolchitosan; Lf: lactoferrin; N,O-CMCS: *N*,*O*-Carboxymethylchitosan-amide conjugate; NLC: nanostructured lipid carrier; NP: nanoparticles; OCS: oligomeric chitosan; PLGA: poly­(lactic-*co*-glycolic) acid; PVA: poly­(vinyl alcohol); SLN: Solid lipid nanoparticle; SPIONs: superparamagnetic iron oxide nanoparticles; RGV: rabbit virus glycoprotein; SiRNA: small interfering RNA; TMC: *N*-trimethylated chitosan.

Most reported applications focus on Parkinson’s and AD, reflecting the limitations of current pharmacological treatments, as previously discussed. In PD, the majority of drugs incorporated into nanoparticles correspond to those already marketed in conventional, non-nanotechnological formulations.^120,123,138,139,152,153,157^ Enhanced nasal mucosal permeation has been demonstrated for several systems, including rotigotine-loaded lecithin–CS nanoparticles,^153^ narigenin-loaded CS nanoparticles,^150^ rasagiline-loaded CS-coated PLGA nanoparticles,^157^ rasagiline-loaded CS glutamate nanoparticles,^158^ and ropinirole-loaded PLGA/CS nanoparticles.^152^ These formulations consistently achieved higher brain concentrations after nasal administration in in vivo studies compared with their conventional counterparts, underscoring the translational potential of CS-based nanocarriers in Parkinson’s therapy (Figure 5).^138,153^

**Figure 5:**
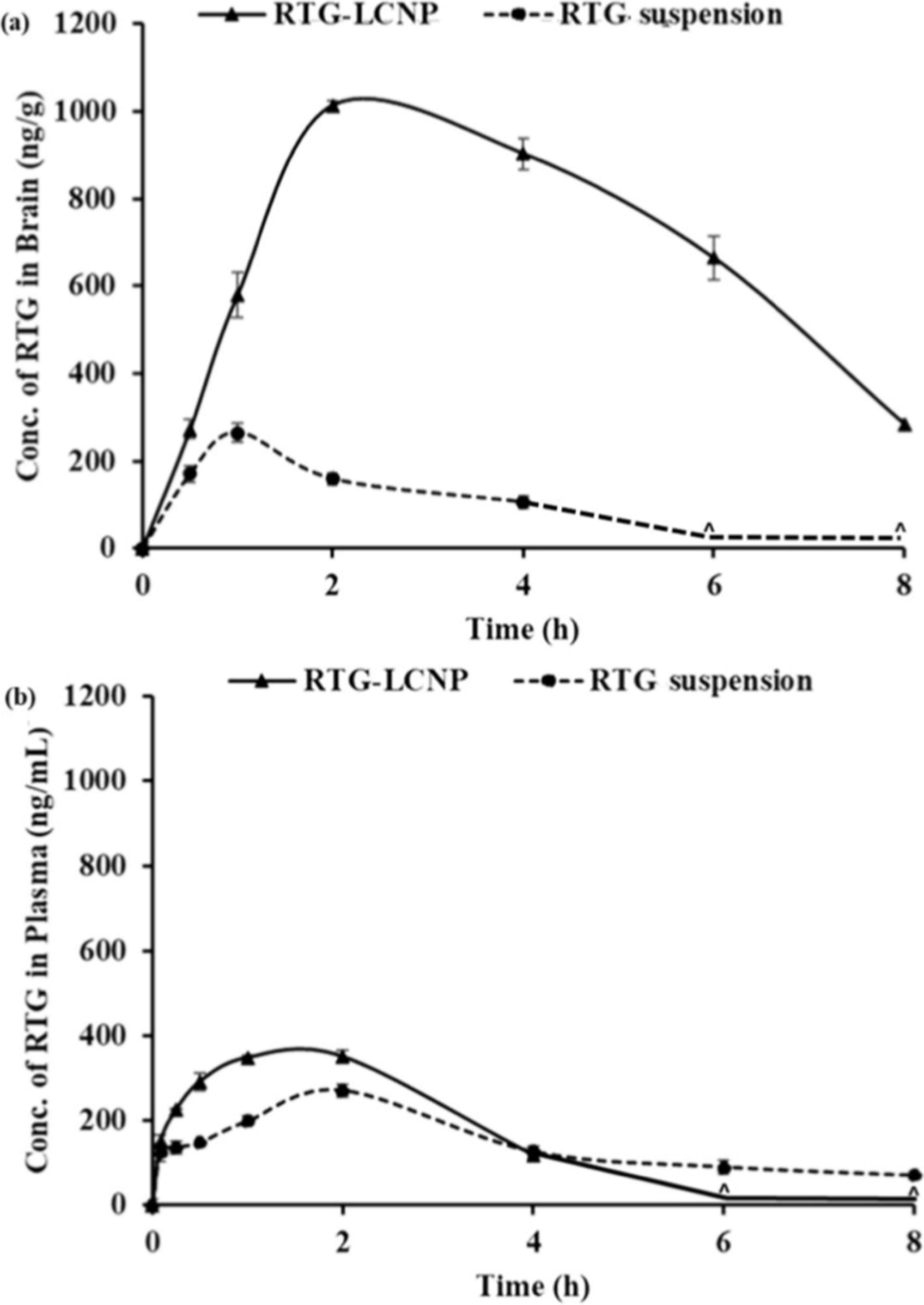
Brain and plasma pharmacokinetic profiles of rotigotine (RTG) following intranasal delivery in male Wister rats (9–10 weeks, 250–260 g). RTG was administered as a suspension and formulated in lecithin–chitosan nanoparticles (RTG-LCNP) at a dose of 2 mg/kg. (a) RTG-LCNP produced markedly higher RTG concentrations in the brain compared with the suspension, indicating superior brain exposure. (b) Corresponding RTG plasma levels. The enhanced brain-to-plasma ratio (a vs b) confirms efficient N2B delivery via the RTG-LCNP formulation. The ^ symbol denotes time points at which RTG concentrations were not detected. Reproduced under the terms and conditions of the Creative Commons Attributions (CC BY) license from Saha et al.].^153^ Copyright [2023], MDPI.

In addition, nanotechnological formulations have demonstrated multiple therapeutic advantages in PD. These include in vivo improvements in motor function,^15,139,148^ increased rats brain dopamine levels, typically reduced in patients with the disorder,^120,156^ and attenuation of oxidative stress through enhanced endogenous antioxidant activity demonstrated in animal studies, which is critical for neuroprotection and slowing disease progression.^148,150^ CS nanoparticles have also been shown to increase nasal mucociliary transport,^138^ thereby prolonging residence time in the nasal cavity of wistar rats,^153^ while complex formation between CS and mucin may further facilitate the uptake of *N*,*O*-carboxymethyl CS nanoparticles in vitro by olfactory ensheathing cells.^123^ Moreover, piribedil-loaded lecithin–CS nanoparticles exhibited increased relative bioavailability in male wistar rats,^138^ along with enhanced N2B transport^148,157,158^ and improved drug-targeting efficiency when compared with conventional, non-nanotechnological formulations.^138,148^

Similarly to nanoparticles developed for PD, CS-based nanoparticles for AD have been shown to enhance nasal mucosal permeation in in vitro, ex vivo, and in vivo studies,^16,159–161^ primarily through improved mucoadhesion^32,106,162,163^ thereby enabling higher drug concentrations to reach the brain.^141,159,162,164^ These pharmacokinetic improvements translated into measurable therapeutic outcomes, including reduced memory impairment in rats^16,140,154,159^ and enhanced long-term memory performance.^16,140^ Consistent with these effects, treated animals also exhibited higher neuronal density, typically diminished in AD.^16^

Furthermore, intranasal administration of CS-based nanoparticles has been shown to improve both N2B distribution and brain-targeting efficiency,^162,165^ while also prolonging cerebral half-life compared with oral administration or conventional drug formulations.^165,166^ For example, the brain half-life of galantamine, an acetylcholinesterase inhibitor clinically used for symptomatic treatment of AD, encapsulated in CS nanoparticles was more than doubled relative to oral administration or intranasal delivery of galantamine solution in a rat model.^149^ Similarly, sinapic acid, a neuroprotective compound involved in synaptic plasticity, loaded into CS nanoparticles achieved a 1.5-fold increase in brain half-life after intranasal administration compared with plain sinapic acid administered orally in BALB/c mice and also demonstrated enhanced neuronal cellular uptake (Figure 6).^106^

**Figure 6:**
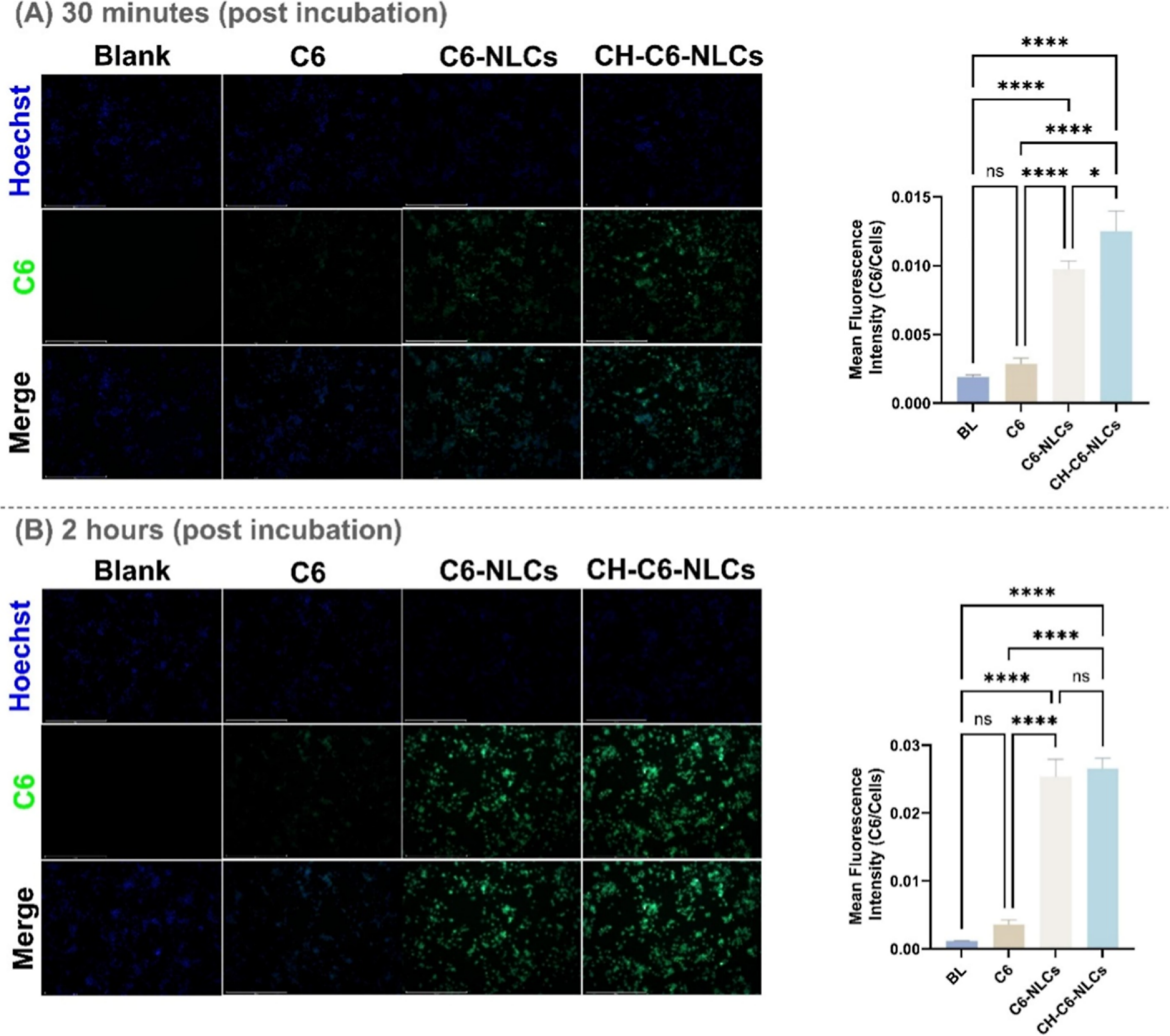
In vitro Cell Uptake in Neuro-2a cells. Representative fluorescence micrographs of cells exposed to blank formulation (BL), free coumarin-6 solution (C6), C6-loaded nanostructured lipid carriers (C6-NLCs), and chitosan-coated C6-NLCs (CH-C6-NLCs) after (A) 30 min and (B) 2 h of incubation (magnification: 10×; scale bar: 500 μm). Data are presented as mean ± SEM (*n* = 3). Statistical comparisons were performed using one-way ANOVA followed by Tukey’s post hoc test (*P < 0.05; ****P < 0.0001). CH-C6-NLCs exhibited markedly enhanced and time-dependent cellular internalization, which is consistent with strengthened electrostatic interactions between the positively charged CH coating and the negatively charged neuronal membrane. Reproduced with permission from [Prabakaran et al.].^106^ Copyright [2025] Elsevier.

Among the molecular targets implicated in AD progression are BACE1, which drives amyloid-β plaque formation; Notch, whose upregulation is associated with neuroinflammation; and Tau and caspase-3, both linked to neuronal loss and disease progression.^133,149,167^ In this context, nanoparticle-based strategies have been investigated to modulate the expression of these targets, either through the encapsulation of siRNA or by delivery of therapeutic small molecules via CS nanocarriers. For instance, BACE1 siRNA was encapsulated in CS-coated solid lipid nanoparticles for N2B administration.^133^ Intranasal delivery of galantamine-loaded CS nanoparticles was reported to downregulate Notch expression, thereby attenuating the neuroinflammation associated with its upregulation.^149^ Similarly, rivastigmine-loaded CS nanoparticles administered intranasally decreased Tau and caspase-3 expression.^167^ In addition, encapsulation of antioxidant small molecules such as narigenin and curcumin reduced oxidative stress in vivo, supporting the notion that antioxidants may serve as complementary strategies to mitigate neuronal damage in AD.^16,141^

Beyond neurodegenerative disorders, CS-based N2B nanocarrier systems have also been investigated in the context of neuropsychiatric conditions, including BD and anxiety.^134^ Although lithium remains the gold standard for BD therapy, fluctuations in its plasma concentration are closely associated with adverse effects. In this regard, intranasal administration of lithium via a sprayable in situ-forming hydrogel composed of chelating oxidized starch nanoparticles and carboxymethyl chitosan was shown to reduce systemic exposure while maintaining therapeutic brain levels, resulting in decreased hyperlocomotion and improved tolerability.^121^ As for anxiety treatment, N2B distribution and brain-targeting efficiency were enhanced with buspirone-loaded CS nanoparticles and buspirone-loaded thiolated CS nanoparticles compared with buspirone solution.^134,145^ Thiolated CS nanoparticles and transferrin-functionalized nanoparticles provided further anxiolytic benefits through improved mucoadhesion and facilitated brain delivery.^124,145^

The application of CS nanoparticles via the N2B route has also shown considerable promise for brain tumor therapy. Studies have encapsulated therapeutic agents within CS-coated nanoparticles to circumvent first-pass metabolism, demonstrating enhanced mucoadhesion and greater drug accumulation in brain tissue of wistar rats.^105^ CS has been specifically selected for its ability to adhere to the nasal mucosa owing to its positive surface charge, which prolongs residence time and enhances drug uptake,^104^ resulting in significant cytotoxicity against glioblastoma cells or measurable tumor regression.

Moreover, synthesis protocols have been increasingly refined to yield nanoparticles with precise control over drug release kinetics and selective activation at the target site, ultimately enhancing therapeutic outcomes. Notably, this design strategy has also been associated with improved antiangiogenic effects,^74,146^ highlighting its relevance for tumor microenvironment modulation. From a translational perspective, CS-based nanocarriers exhibit high nasal permeability and efficient transport to the brain parenchyma, enabling selective accumulation within tumor regions and reinforcing their potential as effective platforms for nose-to-brain drug delivery.^151,168^

Beyond their capacity to enhance brain delivery, CS-based nanoparticles have also emerged as promising platforms for drug repurposing strategies. In particular, simvastatin-loaded CS nanoparticles exhibited notable efficacy against glioblastoma by mitigating hallmark pathological alterations, such as intratumoral hemorrhage, as illustrated in Figure 7.^105^

**Figure 7:**
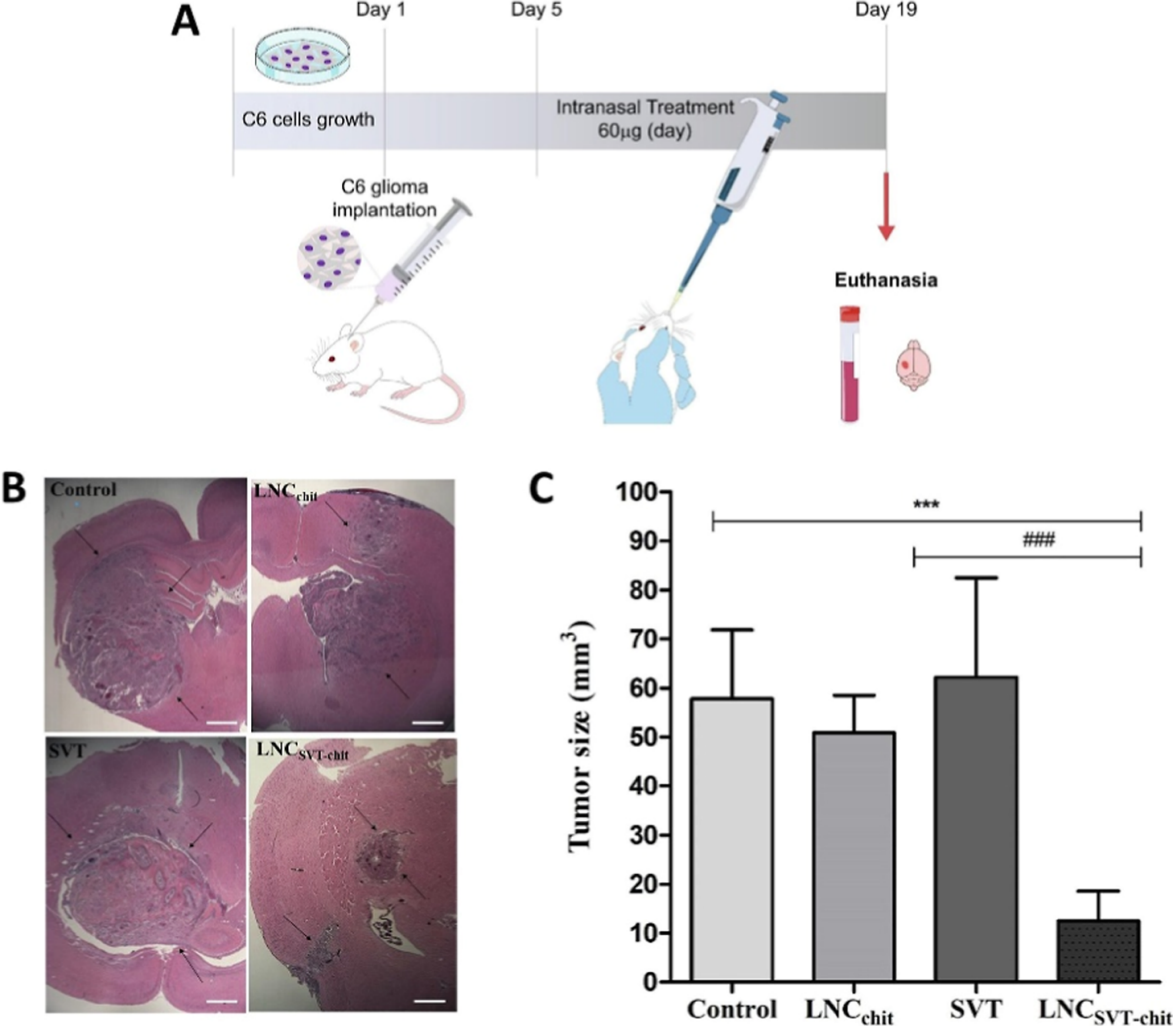
Therapeutic efficacy of chitosan-coated simvastatin-loaded lipid-core nanocapsules (LNCSVT-chit) in a rat glioblastoma model. Male Wistar rats inoculated with C6 glioma cells were intranasally treated once daily for 14 days with vehicle (0.9% NaCl—control), blank LNCchit, free simvastatin (SVT, 60 μg/day), or LNCSVT-chit, 60 μg/day. (A) Schematic representation of the intranasal dosing protocol. (B) Representative hematoxylin and eosin (H&E)-stained brain sections showing glioma after 14 days of treatment. Scale bars = 1 mm. (C) Tumor volume on day 19 (mean ± SD, *n* = 6). LNCSVT-chit treatment resulted in a pronounced reduction in tumor size, whereas free SVT did not significantly affect tumor growth. ***p < 0.001 vs control; ###*p* < 0.001 vs SVT. Reproduced with permission from [Bruinsmann et al.].^105^ Copyright [2022] Elsevier.

In the context of infectious diseases, various therapeutic agents—including antivirals,^169^ antibacterials,^128^ and antiparasitics^170^—have been encapsulated into CS-based nanoparticles for the treatment of neuroAIDS,^169^ cerebral tuberculosis,^128^ and cerebral malaria,^170^ respectively. For instance, CS-coated lipid nanoparticles coloaded with artemether and lumefantrine, a combination of first-line antimalarial drugs, enhanced cerebral drug distribution through improved nasal permeation, resulting in a marked antiplasmodial effect with 95% chemosuppression following intranasal administration.^170^ Similarly, efavirenz, a non-nucleoside reverse transcriptase inhibitor used in HIV therapy, when loaded into CS-g-hydroxypropyl-β-cyclodextrin nanoparticles, exhibited superior nasal permeation due to the increased solubility conferred by β-cyclodextrin grafting, thereby promoting higher N2B delivery.^169^ In addition, spray-dried CS nanoparticles encapsulating rifampicin and isoniazid, both first-line antitubercular drugs, achieved greater area under the curve and prolonged time to maximum concentration compared with oral administration, leading to improved brain distribution and sustained drug release. These pharmacokinetic improvements were directly associated with stronger mycobactericidal effects in murine models of cerebral tuberculosis.^128^

Given the growing body of evidence supporting the efficacy of CS-based NP for N2B delivery, their safety profile must be carefully evaluated. Direct brain deposition via olfactory pathways introduces specific neurotoxicity risks, including microglial activation, neuroinflammation, and potential interference with neuronal signaling. Chronic intranasal administration may also impair olfactory function, increasing the risk of hyposmia or anosmia, highlighting the need for rigorous safety assessment in the development of N2B nanocarriers.^181^ Despite these limitations, this route reduces systemic exposure, potentially lowering off-target toxicity and improving therapeutic outcomes.

Although long-term exposure data remain limited, current evidence suggests a favorable safety profile for intranasal CS-NP. CS-coated formulations consistently have shown lower cytotoxicity than uncoated systems^182^ and CS oligosaccharides have been reported to protect against neuronal damage.^183^ Furthermore, CS-coated NP formulation have been deemed safe for nasal epithelial cell lines (RPMI-2650)^184^ and histomorphological analyses in male Wistar rats have already demonstrated preserved nasal mucosal integrity, with no signs of cellular damage or necrosis following intranasal administration.^185^ Despite these promising findings, few systems have progressed to advanced clinical evaluation, highlighting a key translational bottleneck.

## 5 Patent Review

Over the past decade, a substantial number of patents have been filed on CS-based nanoparticles specifically designed for N2B delivery. For this review, patents were retrieved from three major databases: Espacenet, the World Intellectual Property Organization, and Google Patents. The search strategy combined the keywords “nose-to-brain delivery,” “chitosan nanoparticles,” and either “central nervous system” or “brain.” All of the retrieved entries were individually screened to confirm their relevance to the scope of this review. Table 3 summarizes the identified patents, highlighting key aspects such as formulation characteristics, functional role of CS, active pharmaceutical ingredients, therapeutic indications, and country of origin.

**Table 3:** Patents Related to the Development of CS-Based NP for N2B Delivery in the Treatment of CNS Disorders^a^

patent Number	patent name	Year/Country	formulation	drug	application	ref
WO2016101081A1	NP based on CS for the transport of peptides with activity in the CNS	2016/Chile	CS-basedNP loaded with peptides	Peptides with activities in the CNS	Treatment of CNS disorders	^186^
WO2015063510A1	Delivery of drugs	2016/England	NP consist of an amphiphilic carbohydrate compound (CS and CS-derivatives), a hydrophilic drug (peptide), and one or more pharmaceutically acceptable excipients	Endogenous opioid peptides: leucine-5-enkephalin (LENK) and methionine-5-enkephalin (MENK)	Treatment of brain disorders	^187^
US20180177744A1	Method of treating pain and depression using a hybrid mixture of S-ketamine and R-ketamine	2018/USA	CS-based NP loaded with racemic ketamine	Racemic mixture of R- ketamine (10–30%) and S-ketamine (70–90%)	Treatment of depression and pain	^188^
US10799601B2	Method of making peptide-tagged PEGylated CS nanoparticles	2020/Canada	The NP design entails the chemical modification of a cationic cross-linkable polymer (CS) with a hydrophilic linear polymer (e.g., polyethylene glycol) and a targeting/penetrating peptide for delivering anionic agents	Anionic agents (e.g., RNA, DNA, siRNA, shRNA, miRNA, oDNA, and anticancer drugs	Prevention, treatment, and/or alleviation of symptoms associated with neurodegenerative disease or brain cancer	^189^
WO2017089392A1	Treatment of central nervous tumors	2021/Belgium	CS-NP loaded with siRNA targeting Galectin-1	siRNA targeting Galectin 1	Treatment of central nervous tumors	^190^
BR1020210211458A2	CsS-coated poly(e-caprolactone) NP loaded with sulfamethoxazole and trimethoprim, for i.n. administration, for the treatment of cerebral toxoplasmosis: obtaining process, pharmaceutical composition, and application	2023/Brazil	CsS-coated loaded with sulfamethoxazole and trimethoprim	Sulfamethoxazole and trimethoprim	Treatment of cerebral toxoplasmosis	^191^
CN118021764A	Nanopharmony drug delivery system for active targeting treatment of cognitive dysfunction through nose and brain, and preparation method and application thereof	2024/China	CS-based NPs surface-functionalized with TAT polypeptide and lactoferrin for the delivery of the CCR5 peptide antagonist DAPTA	CCR5 peptide antagonist-DAPTA	Treatment of cognitive dysfunction	^192^
US20240344068Al	Dual function hybrid NP and methods of using the same to treat diseases and disorders	2024/USA	Dual-function hybrid NP consist of CS and siRNA core, surrounded by an outer liposomal layer containing phospholipids and cannabidiol	siRNA and cannabidiol	Treatment of neurological diseases and disorders	^193^

a CS: chitosan; NP: nanoparticles; CNS: central nervous system.

Overall, the patent landscape reveals a rapidly expanding but still fragmented field. Although numerous filings underscore the versatility of CS-based NP as coatings, carriers, or functionalized platforms for N2B delivery, several patents exhibit overlapping claims and incremental modifications, which may limit their differentiation in terms of technological advancement. Most inventions remain anchored in preclinical concepts, with few addressing essential aspects for clinical translation such as reproducible large-scale manufacturing, regulatory compliance, and long-term safety. This gap suggests that while CS nanocarriers are consistently recognized as promising tools for CNS therapy, their patentability often reflects incremental advances rather than disruptive breakthroughs. Future progress hinges on integrating truly disruptive innovations, such as multifunctional systems, improved standardization of CS derivatives, and harmonized regulatory frameworks to effectively bridge the divide between experimental concepts and clinically viable CNS therapies.

## 6 Conclusion and Future Directions

CS-based NP has emerged as one of the most robust and adaptable platforms for N2B drug delivery. A growing body of preclinical evidence supports their capacity to navigate key physiological barriers driven by strong mucoadhesion, transient modulation of tight junctions, and access to the brain via the olfactory and trigeminal pathways. Collectively, these features have translated into encouraging outcomes across a wide range of CNS disorders including neurodegenerative and neuropsychiatric diseases, brain tumors, and infectious conditions, underscoring both the versatility and biological relevance of CS-based systems.

The studies discussed herein demonstrate that CS-based NP enhances N2B drug delivery by improving permeation, mucoadhesion, and residence time in the nasal mucosa, thereby increasing targeting efficiency and brain exposure. These systems facilitate transport to the brain parenchyma, enhance neuronal uptake, and enable sustained drug release, resulting in a prolonged cerebral half-life while reducing systemic exposure. Collectively, these effects have been associated with improved pharmacological outcomes, supporting their potential as versatile and effective platforms for the treatment of CNS disorders. Despite this promise, however, clinical translation remains limited by unresolved challenges related to manufacturing scalability, long-term safety, and regulatory alignment, highlighting the need for continued innovation and coordinated multidisciplinary efforts.

One of the most persistent bottlenecks lies in the lack of scalable, reproducible, and clinically compliant production strategies. Widely used fabrication methods, such as ionic gelation, remain largely restricted to small-batch laboratory settings. In this context, microfluidic technologies offer an attractive bridge between bench-scale innovation and industrial manufacturing, enabling continuous production with precise control over the particle size and surface properties. Nevertheless, their implementation at a scale introduces new hurdles, including device fouling, microfabrication costs, and stringent Good Manufacturing Practice requirements. Addressing these challenges will require robust process control, validation strategies, and early regulatory engagement.

Translational hurdles are further compounded by anatomical and physiological differences between commonly used rodent models and the human nasal cavity, particularly with respect to epithelial organization, surface area, and mucus composition. Although advances in in vitro and ex vivo nasal models have improved our mechanistic understanding, their predictive power remains limited. The adoption of more physiologically relevant platforms, such as organ-on-a-chip systems and large-animal models including sheep or nonhuman primates, will be essential to strengthen translational relevance and better anticipate human outcomes.

At the formulation level, the performance of CS-based NP is highly sensitive to parameters such as the polymer molecular weight, degree of deacetylation, particle size, and surface charge. A deeper, mechanistic understanding of how these variables influence interactions with nasal mucus, epithelial barriers, and neuronal pathways is still lacking. Emerging tools in high-resolution imaging, computational modeling, and single-cell transcriptomics offer new opportunities to elucidate these complex interactions. In parallel, machine learning and AI-driven approaches may accelerate rational formulation design by identifying optimal parameter spaces from increasingly large and heterogeneous data sets.

While the chemical versatility of CS enables surface modification and ligand-mediated targeting, important questions remain regarding the long-term safety and immunogenicity of modified systems, particularly under chronic dosing conditions. In addition, the inherent variability of natural polymers raises concerns about batch-to-batch consistency, reinforcing the need for rigorous quality control and validated analytical frameworks. Furthermore, extended repeat-dose toxicity studies are essential to facilitate regulatory approval and clinical translation. The development of regulatory pathways specifically tailored to N2B nanomedicines, building on emerging guidance from agencies such as the FDA and EMA, will be critical to derisk clinical development and ensure patient safety.

Beyond conventional drug delivery, the full potential of CS-based NP remains largely untapped in areas such as gene therapy, modulation of neuroinflammation, and theranostic applications. Future efforts should prioritize multifunctional platforms capable of integrating therapeutic delivery, targeting, and real-time monitoring within a single system. Advances in engineering strategies that combine precise nanoparticle design with scalable manufacturing will be pivotal in translating these concepts from a proof-of-concept to clinical reality.

Ultimately, progress in N2B drug delivery will depend on a deeper convergence of materials science, neuroscience, and clinical pharmacology. Designing more sophisticated carriers alone would not be sufficient. A patient-centered perspective, accounting for interindividual variability in disease biology, nasal anatomy, and treatment adherence, must guide future development. In this broader context, CS-based NP represent more than delivery vehicles; they offer a transformative platform with the potential to reshape therapeutic paradigms across neurological, oncological, and psychiatric disorders. Realizing this potential will require sustained innovation, seamless integration between preclinical and clinical research, scalable, and standardized manufacturing strategies and a firm commitment to personalized medicine.

## Abbreviations

AD: Alzheimer’s disease
ADHD: attention-deficit/hyperactivity disorder
BBB: blood–brain barrier
BD: bipolar disorder
BDNF: brain-derived neurotrophic factor
CMCh: carboxymethyl chitosan
CNS: central nervous system
COMT: catechol-*O*-methyltransferase
CS: chitosan
EMA: European medicines agency
FDA: Food and Drug Administration
GABA: gamma-aminobutyric acid
GBM: glioblastoma multiforme
GCPQ: palmitoyl glycol chitosan
IL-6: interleukin-6
MAOIs: monoamine oxidase inhibitors
MDD: major depressive disorder
N: O-CMCS, N,O-carboxymethyl chitosan
N2B: nose to brain
NLC: nanostructured lipid carrier
NMDA: *N*-methyl-d-aspartate
NP: nanoparticles
OCS: oligomeric chitosan
PD: Parkinson’s disease
PLGA: poly­(lactic-*co*-glycolic) acid
PVA: poly­(vinyl alcohol)
SCZ: schizophrenia
SLN: solid lipid nanoparticle
SNRIs: serotonin–norepinephrine reuptake inhibitors
SPIONs: superparamagnetic iron oxide nanoparticles
SSRIs: selective serotonin reuptake inhibitors
TMC: *N*-trimethylated chitosan
TMZ: Temozolomide
TNF-α: tumor necrosis factor-alpha
VEGF: vascular endothelial growth factor
WHO: World Health Organization
WIPO: World Intellectual Property Organization
